# SWAP-70: A New Type of Oncogene

**DOI:** 10.1371/journal.pone.0059245

**Published:** 2013-03-12

**Authors:** Chung-Li Shu, Jing-Yang-Lai, Liang-Cheng Su, Chih-Pin Chuu, Yasuhisa Fukui

**Affiliations:** Institute of Cellular and System Medicine, National Health Research Institutes, Zhunan Town, Miaoli County, Taiwan, Republic of China; University of Chicago, United States of America

## Abstract

SWAP-70 is a protein that has been suggested to be involved in regulation of actin rearrangement. Having discovered that an artificially-derived mutant of SWAP-70 can transform mouse embryo fibroblasts, we searched for naturally-occurring mutations in the SWAP-70 gene, finding listings for several on the Web at www.sanger.ac.uk/genetics/CGP/cosmic/, including three mutations found in ovarian cancers. (The number of such mutations has now reached 13 out of 228 tumors). We created expression vectors for the mutant SWAP-70 proteins and introduced these into NIH3T3 cells. The cells expressing the mutant SWAP-70 constructs exhibited faster growth than the parental or wild-type SWAP-70-expressing cells. In most instances, cells that are able to grow in soft agar will form tumors in nude mice. While SWAP-70-transformed cells grew in soft agar, they failed to form tumors in nude mice. This result implies that transformation by the SWAP-70 mutants is unique. The cells bearing the mutant SWAP-70 genes were sensitive to nutrient starvation, supporting the idea that they are transformed cells. However, they failed to pile up and demonstrated contact inhibition, unlike most normal transformed cells. Upon expression of human SWAP-70 genes, MEK1 was activated. This activation appeared to contribute to the saturation density of the cells. As SWAP-70 has been shown to be the last protein to receive signals from cytokines, it is likely that there is a putative feedback signaling pathway, and that disorder of this signaling pathway can transform cells. Accordingly, this may explain why SWAP-70-transformed cells have different characteristics than most transformed cells.

## Introduction

SWAP-70 is a phosphatodylinotsitol trisphophate (PIP_3_) binding protein involved in actin rearrangement [1]–[3]. It has an EF-hand-like domain at the amino terminal portion and a PH domain at the center of the protein, which is responsible for PIP_3_ binding activity [2], [4]. The EF-hand-like domain may contribute to binding activated Rac1 [4]. Most of the remaining parts of SWAP-70 comprise a coiled-coil structure. In addition, an F-actin binding domain resides at the very-carboxyl terminal end of SWAP-70 [1]. SWAP-70 is abundantly expressed in B cells, however it is almost ubiquitously expressed at low levels [5]. SWAP-70 has been shown to be involved in regulation of actin rearrangement. For example, SWAP-70 is important for homing of B cells [3], which may be the result its role in actin rearrangement. In adherent cells, SWAP-70 resides in the cytosol. In Cos7 cells, upon stimulation with EGF, SWAP-70 moves to the plasma membrane and accumulates at membrane ruffles, suggesting that this protein is important for regulation of membrane ruffling [6]. Indeed, kidney cells cultured from SWAP-70 deficient mice exhibit impaired membrane ruffling after EGF stimulation [2]. Since SWAP-70 binds to PIP_3_ (a component of the plasma membrane), to activated Rac1 (which has been suggested to cause membrane ruffling), and to F-actin (which is a driving force in membrane ruffling), this protein likely plays an important role in the regulation of membrane ruffling. This process is also related to actin rearrangement.

On the other hand, we have noticed that SWAP-70 knockout mouse embryo fibroblasts (MEFs) grow more slowly than wild-type MEFs [7]. MEFs transformed by the src oncogene grow quickly; but those lacking SWAP-70 grow more slowly than counterpart wild-type transformants [7]. MEFs transformed by the src oncogene readily form colonies in soft agar; but those lacking SWAP-70 fail to do so [7]. Most importantly, a mutant form of SWAP-70, which resides at the plasma membrane, was able to transform MEF cells without any stimulation [8]. The transformants grow faster than the wild-type cells, are sensitive to nutrient starvation, require less serum, and are able to grow in soft agar. These results suggest that SWAP-70 can act as an oncogene.

In this paper, we describe mutant SWAP-70 genes found in human tumors, which can transform NIH3T3 cells in a unique fashion, suggesting that SWAP-70 is a novel type of oncogene in humans. SWAP-70 has been shown to be the last protein to receive signals from cytokines. All oncogenes found to date are upstream factors that regulate cell growth signaling or are transcription factors that regulate expression of proteins in a manner important for cell growth. However, the results of the current study indicate that there may be putative feedback signaling from the terminus of these signals, and that this feedback signaling can contribute to causing cancer.

## Materials and Methods

### Cells and culture conditions

NIH3T3 cells were cultured in Dulbecco's modified minimal essential medium (DMEM) – high glucose (4500 mg/L) supplemented with 4 mM L-glutamine and 10% calf serum.

### Establishment of cell lines carrying the exogenous SWAP-70 genes

To obtain MEF clones expressing human mutant SWAP-70, an expression vector pFLAG-C1 harboring wild-type or mutant SWAP-70 was used. pFLAG-C1 was produced by substituting the GFP gene of pEGFP-C1 (GE Healthcare, London, UK) with the sequence for FLAG tag. The point mutations found in the cancer tissues were introduced by PCR. 20 µg of DNA was introduced into about 3×10^6^ cells by electroporation using a Gene Pulser (Bethesda Research Laboratories, Bethesda, MD, USA) at 215 V with 800 µF capacitance. Stable cell lines were established by selection with 300 µg/ml G418 (Merc-Millopre, Darmstadt, Germany), followed by the cloning of colonies formed under these conditions.

### Antibodies and Western blotting

Anti-ERK1/2 and anti-phospho-ERK1/2 antibodies were purchased from Cell Signaling Technology Inc. (Danvers, MA, USA). Anti-FLAG and Anti-β-actin antibodies were from Sigma-Aldrich (St. Louis, MO, USA). Western blotting was performed as previously described. Briefly, blocking was done with 1% skim milk in Tris-buffered saline (TBS)-0.1% Tween20; and the protein was visualized using the ECL system.

### Soft agar colony formation assay

1×10^4^ cells were suspended in 2 ml of a medium containing 0.2% agarose and plated onto a basal agarose layer consisting of a medium containing 0.5% agarose in 6-well plates and incubated for 2 weeks. 2 ml of a medium containing 0.2% agarose were added onto the culture after a week.

### Determination of cell growth

Cells were plated on a 6-cm dish at a density of 1×10^5^ per dish and maintained by changing medium every other day. The number of cells per dish was counted. For examination of cell survival, cells were plated at a density of 2×10^5^ cells per 6-cm dish and maintained without changing medium. The counts are the means of three independent experiments. PD98059 and U0126 were purchased from Wako, Tokyo, Japan.

### Serum requirement for cell growth

Cells were plated at a density of 2×10^5^ cells per 6-cm dish and cultured for 6 days in medium containing 1% serum, with a medium change on day 2.

## Results

### Mutations are found in human cancers

**Figure 1:**
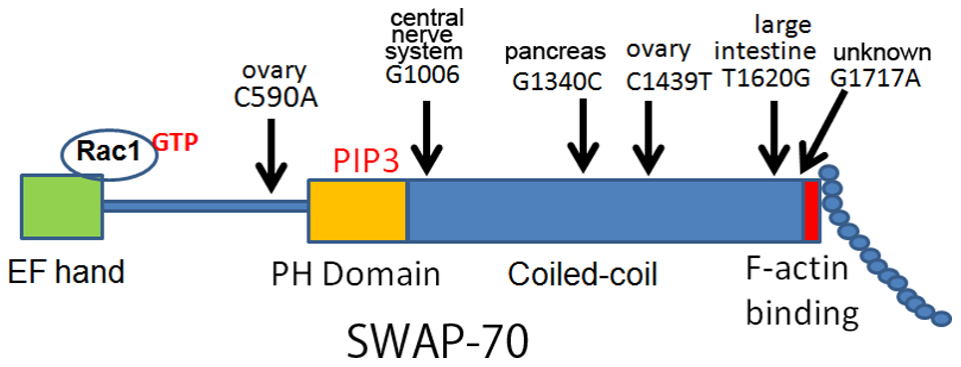
SWAP-70 gene and the mutations found in human tumors. The SWAP-70 coding region is shown in the middle. It has an EF-hand-like domain, which contributes to binding of activated Rac1. In the center portion, the protein has a PH domain that is responsible for PIP_3_ binding activity. This domain is followed by a long coiled-coil domain, which may contribute to the formation of dimers or trimers. At the very-carboxyl terminal end, it has an F-actin binding domain. This region may be masked in the normal conformation; but upon activation, it may become exposed. The arrows show the positions of mutations. The amino acid changes are shown by single letters. The organs are also shown.

As mentioned above, the fact that an artificial mutant form of SWAP-70 transforms MEFs suggests that SWAP-70 can act as an oncogene. We therefore searched for mutations in human cancer. The Web site at http://www.sanger.ac.uk/genetics/CGP/cosmic/supplies such information. At the time we began our experiments, the Web site exhibited three mutations in cancer (Fig. 1): SWAP70-590, SWAP-70-1006, and SWAP-70-1439, named according to the positions of the mutations. Two of these, SWAP-70-1006 and SWAP-70-1439, reside in the coiled-coil domain, and their individual expression led to similar phenotypes, as described below. We created expression vectors encoding the SWAP-70 gene bearing the mutations mentioned above. To distinguish the mutant from the endogenous SWAP-70 gene, a FLAG tag was attached at the amino-terminal end. As addition of GFP to the amino-terminal end does not harm the activity of SWAP-70 [2], this suggests the addition of a FLAG tag should also not harm the activity of SWAP-70.

### Mutant SWAP-70 can transform NIH3T3 cells

**Figure 2:**
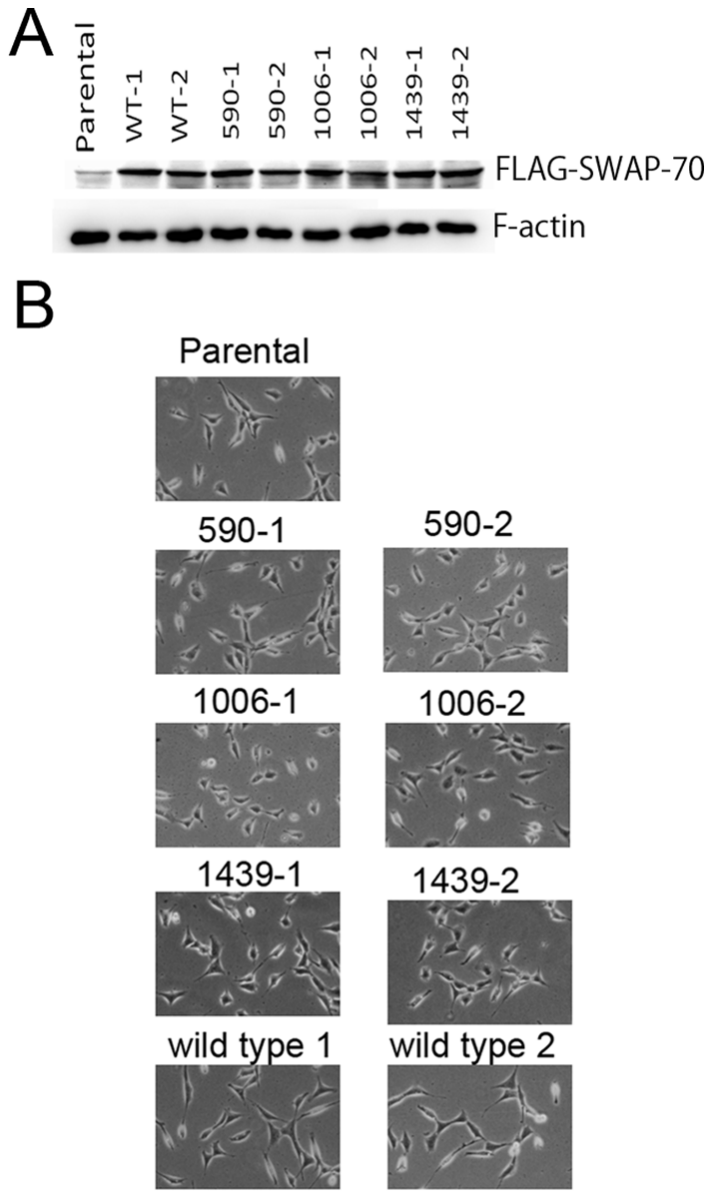
Expression of mutant SWAP70 s and morphology of the transfectants. A, Expression of FLAG-tagged SWAP-70 was detected by Western blotting with an anti-FLAG antibody. B, Morphology of the transfectants. The cells expressing mutant SWAP-70 s were slightly shorter than the parental or the wild-type SWAP-70-expressing cells.

**Figure 3:**
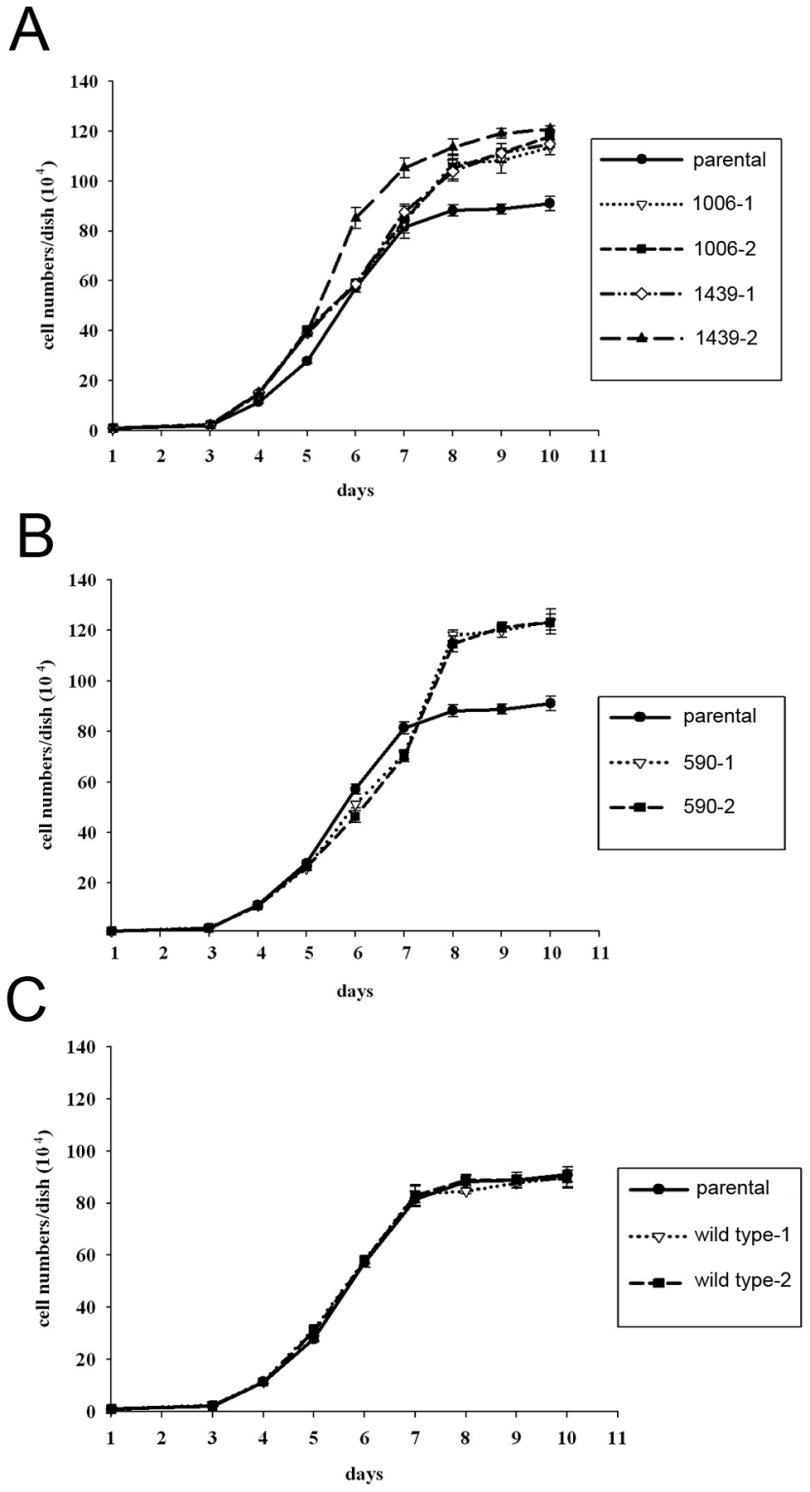
Growth curve of the cells expressing mutant SWAP-70 s. 1×10^5^ cells were plated onto 6-cm dishes, and cell numbers were counted every day. The medium was changed every other day. For reference, the growth curves of the parental cells are shown in every graph. The symbols for the cell lines are shown in the right-hand boxes.

A focus-formation assay using NIH3T3 cells did not reveal any foci. To see the effect of expression of the mutant SWAP-70 gene, we obtained mutant SWAP-70-expressing cells by G418 selection. As a result, we obtained several clones expressing the mutant SWAP-70 s (Fig. 2). In these clones, cell length was slightly shorter than the parental cells (Fig. 2). Expression of wild-type SWAP-70 did not affect the morphology of the cells. The colonies formed by cells expressing the mutant SWAP-70 s were bigger than those formed by introduction of wild-type SWAP-70, suggesting that mutants grew slightly faster than the parental cells. Expression of wild-type SWAP-70 did not significantly enhance cell growth. The growth curves of cells expressing exogenous SWAP-70 are shown in Fig. 3. The cells harboring SWAP-70 with mutations within the coiled-coil domain, SWAP-70-1006 and SWAP-70-1439, exhibited similar growth curves. They grew faster and had higher saturation densities than the parental cells. In contrast, cells expressing SWAP-70-590 grew more slowly than the parental cells at the beginning. Later, however, the growth rate increased, gradually becoming faster than that of the parental cells. Moreover, SWAP-70-590 cell density reached the highest level of all the clones we obtained. In any case, the mutant SWAP-70-harboring cells grew more aggressively than the parental cells, although they maintained contact inhibition and never piled up. This is likely why we failed to observe foci in a focus formation assay. The wild-type SWAP-70 enhanced cell growth only slightly.

**Figure 4:**
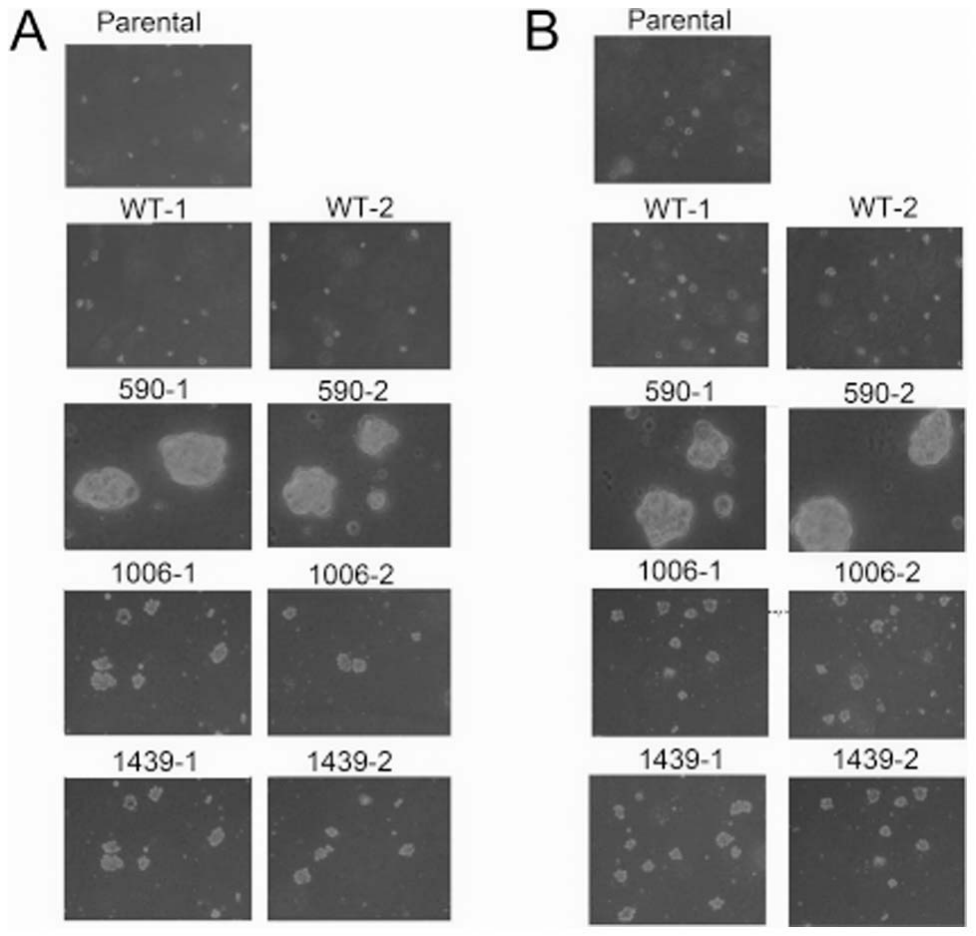
Soft agar colony formation of the cells expressing mutant SWAP-70 s. (A) 1×10^4^ cells were plated into soft agar in 6-well plates. Cells were observed for 10 days. On the sixth day, agar overlay was performed. (B) Similar experiments were performed in the presence of 10 µM PD98059.

To further examine the characteristics of the mutant SWAP-70-harboring cells, a soft agar colony formation assay was performed. The parental clone did not grow in the soft agar. However, cells expressing SWAP-70-590 grew well within the soft agar (Fig. 4A). In contrast, cells expressing SWAP-70-1006 or SWAP-70-1439 formed small colonies (Fig. 4A). In most cases, cells capable of forming colonies in soft agar can form tumors in nude mice. However, none of our SWAP-70 mutant clones formed tumors in nude mice (data not shown).

**Figure 5:**
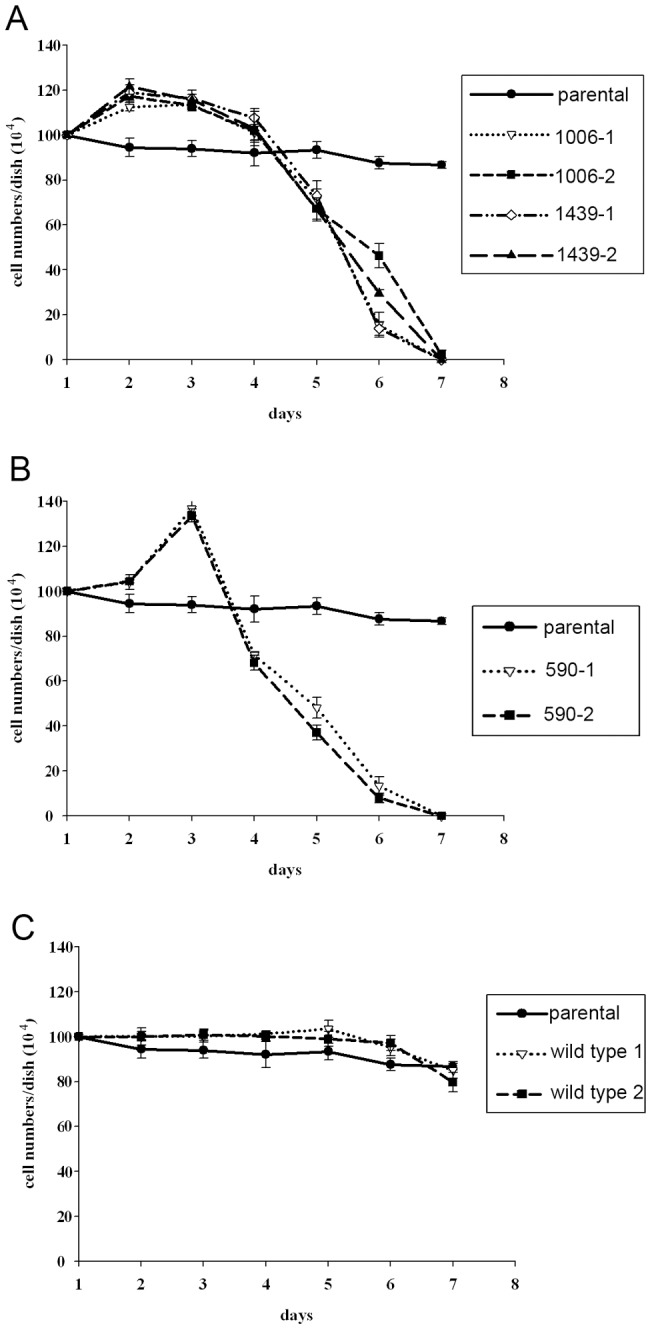
Effect of nutrient starvation on the cells expressing mutant SWAP-70 s. 1×10^6^ cells were plated onto 6-cm plates, and cell number was counted every day. Medium was not changed during the experiments. For reference, the growth curves of the parental cells are shown in every graph.

Transformed cells are often sensitive to nutrient starvation, reflecting the fact that they cannot stop their metabolism, and continue to grow even when nutrients are exhausted. Cells were plated at the saturation density of the parental cells and cultured without changing medium. The parental cells remained alive for a week; however, cells expressing mutant SWAP-70 showed a small amount of growth and then died, which suggests that these cells cannot stop growing and continue to consume nutrients (Fig. 5). This is one of the characteristics of transformed cells.

These results suggest that mutant SWAP-70-expressing cells are transformed even though they fail to pile up and maintain contact inhibition.

### Abnormal characteristics of the mutant SWAP-70 expressing cells as compared to most transformed cells

**Figure 6:**
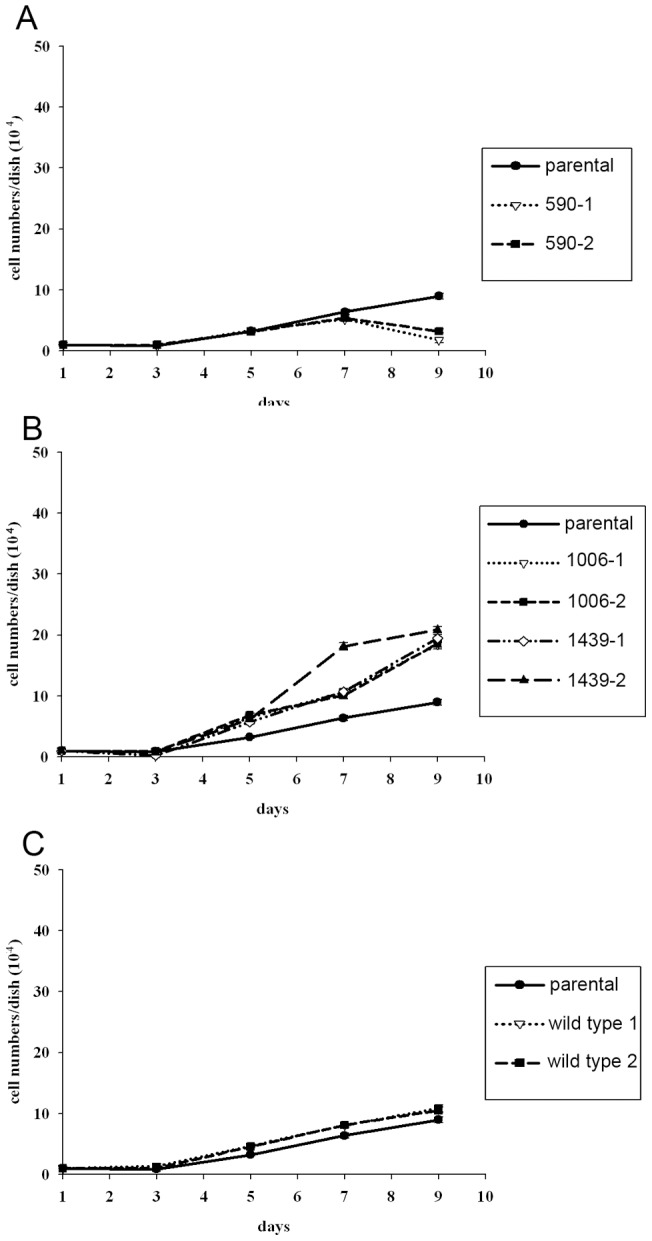
Growth of cells expressing mutant SWAP-70 under low serum conditions. 2×10^5^ cells were plated onto 6-cm dishes in the medium containing 1% calf serum, and cell numbers were counted every other day. The medium was exchanged every other day. For reference, the growth curves of the parental cells are shown in every graph. The symbols for the cell lines are shown in the right-hand boxes.

In most cases, transformed cells require less serum for growth. Therefore, we tested the dependency on serum of our transformed lines (Fig. 6). The parental cells grew slowly. The cells expressing a mutant coiled-coil domain grew faster than the parental cell lines. However, cells expressing SWAP-70-590 reacted differently. Their growth was retarded, until finally, they died. This unusual result indicates that the transformation of cells by SWAP-70 is not a typical one.

### Expression of human SWAP-70 in NIH3T3 cells activates MEK

**Figure 7:**
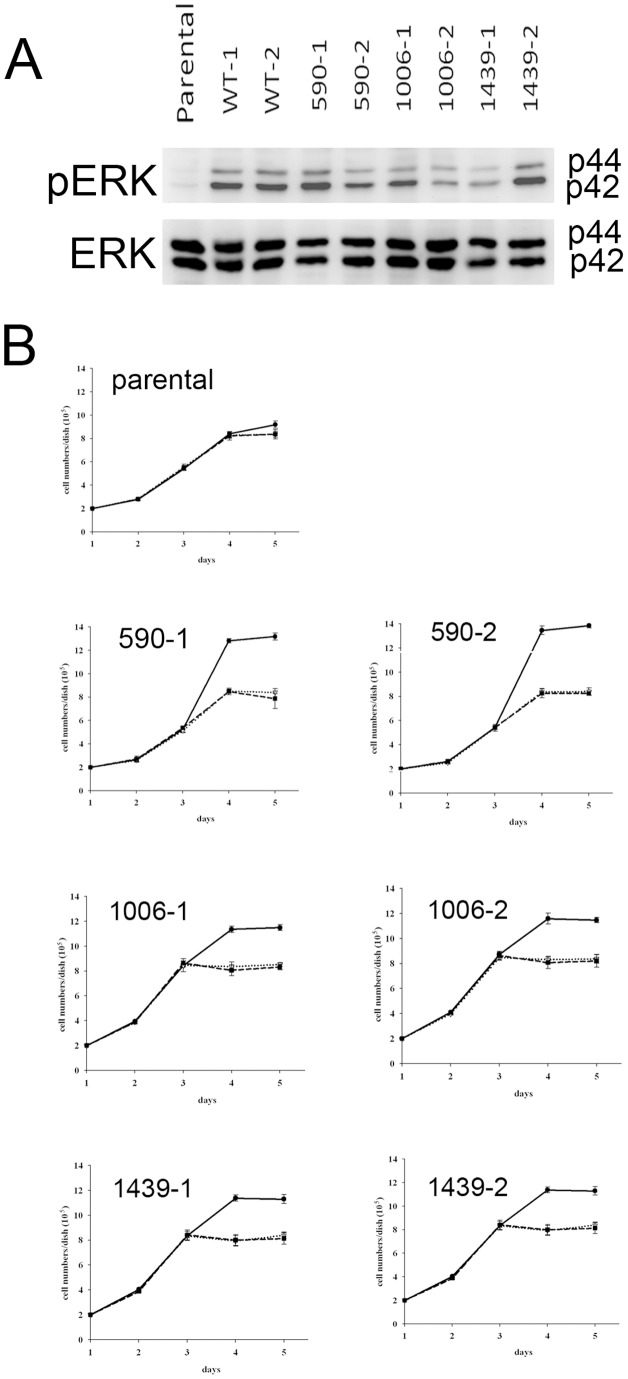
Activation of Erk after expression of human SWAP70 in NIH3T3 cells and its effect on the growth of the cells. A, Activation of MEK was examined by phosphorylation of Erk, p42, and p44. The assay was done by Western blotting with anti-Erk or anti-phospho-Erk. B, 1×10^5^ cells were plated onto 6-cm dishes, and cell numbers were counted every day. Medium was changed every other day. The dotted and broken lines show growth curves when 10 µM U0126 and 10 µM PD98059 were added to the medium, respectively.

A previous report using mouse embryonic fibroblasts suggests that activation of MEK1 may be related to expression of SWAP-70 [8]. As shown in Fig. 7A, expression of human SWAP-70 (even the wild-type form) activated MEK1. This result implies that mouse and human SWAP-70 functions may have species-specific differences. Activation of MEK1 did not affect the growth rate of NIH3T3 cells. However inhibition of MEK1 by specific inhibitors affected the saturation densities of the cells transformed by the mutant SWAP-70 (Fig. 7B). Mutant saturation densities dropped to a level similar to that of the parental cell line. These results suggest that activation of MEK1 may play a role in increasing the saturation density of mutant SWAP-70-transformed cells.

To test whether activation of MEK1 affects malignancy of the transformed cells, a MEK1 inhibitor, PD98059, was added to the soft agar colony formation assay. However, we saw no effects in any cell lines, suggesting that activation of MEK1 does not affect growth in soft agar (Fig. 4B).

## Discussion

In this study, we found that SWAP-70 mutations found in human cancer can transform mouse NIH3T3 cells. SWAP-70 is the protein that lies most downstream of the signal transduction of growth factors and regulates actin rearrangement. Most oncogenes code for the proteins that regulate signaling for cell growth. However, SWAP-70 has no downstream factors to activate. We propose that there may be a putative feedback signaling pathway from the terminus of the signaling and that disorder of this signaling can result in the formation of tumors. This idea could open up new approaches to the study of tumor formation.

Because the signaling pathway affected by SWAP-70 mutations may be an unknown feedback mechanism, phenotypes of the transformed cells were somewhat abnormal or incomplete. The cells expressing SWAP-70-590 formed large colonies in soft agar, grew faster than the parental cell line, and were sensitive to nutrient starvation but required a high concentration of serum and failed to form tumors in nude mice. The cells expressing coiled-coil domain mutants grew faster than the parental cell line, formed small but significant colonies in soft agar, and grew under low-serum conditions, but failed to form tumors in nude mice. In most cases, cells capable of growing in soft agar form tumors in nude mice. For some time now, the soft agar colony formation assay has been believed to be a model assay to reveal the malignancy of tumor cells. This is because there is a good correlation between tumor formation in animals and soft agar colony formation. Therefore, our data appears to be quite abnormal. These results might be related to the fact that SWAP-70-transformed cells maintained contact inhibition. This may suggest that the signaling related to SWAP-70-dependent transformation of cells is not a growth-stimulating signal-transduction pathway but rather an unknown feedback-signaling mechanism. However, an overall consideration of all phenotypes suggests that mutations in the SWAP-70 gene could contribute to the formation of tumors. Increased growth rate, high saturation density, and sensitivity to nutrient starvation are typical characteristics of transformed cells. We suggest that because the pathway that is disordered by mutant SWAP-70 is not a growth-stimulating pathway, the phenotype of transformation is different from the conventional one, which is caused by the activation of growth-stimulating pathways.

It is surprising that expression of human SWAP-70 activated MEK1. Even wild-type SWAP-70 activated MEK1. However, MEK1 activated by wild-type SWAP-70 did not have any effect on growth properties of the cells. MEK1 activated by mutant SWAP-70 did not enhance cell growth, but may have contributed to higher saturation density of the cells since inhibition of MEK1 by specific inhibitors lowered saturation densities. After contact inhibition, transformed cells appeared denser than control cells. It is possible that the attachment capabilities of transformed cells to the substratum were weaker than in wild-type cells, allowing transformed cells to push up a little to occupy less area, and inhibition of MEK may restore the attachment capabilities close to the parental cells. This may make the saturation densities of the MEK-inhibited transformed lower cells than those of the cells without inhibition of MEK. Even though transformed cells did not grow on top of each other, these results revealed an increased growth capacity compared to control cells. Inhibition of MEK did not affect cell growth in soft agar. This suggests that activation of MEK1 was independent of the mechanism increasing growth rate.

In this paper, we propose (1) that SWAP-70 is a novel type of oncogene that affects feedback signaling and (2) that feedback signaling can contribute to regulation of cell growth.
